# Supplementary material for: Comparative genomics provides new insights into the diversity, physiology, and sexuality of the only industrially exploited tremellomycete: Phaffia rhodozyma
Source: BMC Genomics. 2016 Nov 9;17:901. doi: 10.1186/s12864-016-3244-7 (PMC5103461; doi:10.1186/s12864-016-3244-7)
Supplement: Additional file 6: — List of orphan genes with links to PFAM (related to Additional file 1: Table S1). (ZIP 1428 kb) [file 12864_2016_3244_MOESM6_ESM.zip › PFAM_UNIQUE/G00410_P.html]

#                                                                            --- full sequence --- -------------- this domain -------------   hmm coord   ali coord   env coord
  
# target name        accession   tlen query name           accession   qlen   E-value  score  bias   #  of  c-Evalue  i-Evalue  score  bias  from    to  from    to  from    to  acc description of target
  
#------------------- ---------- ----- -------------------- ---------- ----- --------- ------ ----- --- --- --------- --------- ------ ----- ----- ----- ----- ----- ----- ----- ---- ---------------------
  
G00410\_P             -            215 Dsh\_C                PF12316.3    203       2.3    6.9   5.6   1   1    0.0018       1.1    8.0   3.3    34   144    89   203    84   211 0.43 - Segment polarity protein dishevelled (Dsh) C terminal  
G00410\_P             -            215 DUF4592              PF15262.1    130         5    6.4   9.0   1   2      0.31   9.2e+02   -1.0   2.2    82    87    73    78    33   131 0.55 - Domain of unknown function (DUF4592)  
G00410\_P             -            215 DUF4592              PF15262.1    130         5    6.4   9.0   2   2   0.00016      0.47    9.7   2.4    20    87   128   194   118   204 0.76 - Domain of unknown function (DUF4592)  
G00410\_P             -            215 NUDE\_C               PF04880.8    166         2    7.4   8.1   1   1     0.003       2.3    7.2   7.3    55   134    45   137    36   143 0.83 - NUDE protein, C-terminal conserved region
